# The effect of acupuncture on postpartum stress urinary incontinence: A protocol for systemic review and meta-analysis

**DOI:** 10.1097/MD.0000000000029177

**Published:** 2022-07-22

**Authors:** Fengye Cao, Shanshan Zhang, Jingmei Huang, Lin Gan, Qinshuai Zhuansun, Xianming Lin

**Affiliations:** a The Third Clinical Medical College of Zhejiang Chinese Medical University, Hangzhou City, Zhejiang Province, China; b The Second Affiliated Hospital of Zhejiang Chinese Medical University, Hangzhou City, Zhejiang Province, China.

**Keywords:** acupuncture, meta-analysis, postpartum, protocol, stress urinary incontinence

## Abstract

Postpartum stress urinary incontinence (PSUI) is a widespread complaint in postpartum women, which significantly affects their quality of life. Acupuncture has been widely used as an alternative complementary therapy for the treatment of PSUI. This protocol is carried out to comprehensively explore the effectiveness and safety of acupuncture for treating PSUI.

**Methods:**

Randomized clinical trials related to acupuncture treatment of PSUI will be searched in Chinese and English literature databases: PubMed, Web of Science, EMBASE, Cochrane Central Register of Controlled Trials, Chinese National Knowledge Infrastructure, Chinese Biomedical Literature Database, Wanfang Database, and the Technology Periodical Database. Changes in pelvic floor muscle strength compared with baseline will be accepted as the primary outcomes, and secondary outcomes will be the International Consultation on Incontinence Questionnaire-Urinary Incontinence Short Form score, the urodynamic indexes, the incontinence quality of life questionnaire, and adverse effects of acupuncture. All publications will be screened and extracted by 2 reviewers independently. Quality of the eligible publications will be assessed according to the Cochrane Risk of Bias tool and statistical analyses will be conducted by using the Review Manager V.5.3.

**Results:**

This study will provide a high-quality comprehensive evaluation for the clinical efficacy and safety of acupuncture for PSUI.

**Conclusion:**

This systematic review will provide comprehensive evidence of acupuncture treatment on specific outcomes for PSUI.

**Ethics and Dissemination:**

Because of the study will not collect personal information, ethical approval will not be required. The results will be published in a peer-reviewed journal.

**Trial registration:**

INPLASY 202220045.

## 1. Introduction

Stress urinary incontinence, a phenomenon of urinary involuntary overflow when abdominal pressure suddenly rises, is the most type of urinary incontinence in postpartum women.^[1,2]^ Although most women will alleviate the symptoms of incontinence within 3 months after the birth, a significant percentage of them cannot recover during the postpartum period and eventually develop persistent urinary incontinence.^[3–5]^ Current researches have shown that labor injuries such as vaginal spontaneous delivery and vacuum or forceps delivery are considered to greatly increase the incidence of postpartum stress urinary incontinence (PSUI).^[6,7]^ PSUI is not a life-threatening disease, but it greatly reduces the life quality of puerperae and limits their daily activities.^[8,9]^ More importantly, PSUI can seriously affect emotions, leading to postpartum psychological disorders such as low self-esteem, anxiety and depression.^[10–12]^

The main treatments for PSUI include supervised pelvic physical floor therapy (PFMT), medications and surgery.^[3,13]^ Though effective, these approaches show multiple limitations. The most frequently prescribed drug for PSUI remains to be α-adrenergic agonists such as midodrine hydrochloride, which also caused significant intolerable side effects, including hypertension, urinary retention, and paresthesia. A large number of women initially provided drug therapy will eventually received surgery for their incontinence. Surgery is a significant approach. However, complications of surgical treatments are inevitable, such as bladder injury, urinary dysfunction, and infection, etc.^[14–16]^ It is reported that PFMT is more effective than no treatment, placebo drug or inactive control treatments for women with PSUI. Unfortunately, in a 15-year follow-up, long-term adherence to PFME was found to be low, with no difference between intensive and home training programs.^[17]^ In summary, there is a clinically unmet need and a mandate for effective, lower cost, noninvasive treatment, especially for people living in low-income regions.

As one of the important methods of Traditional Chinese Medicine , acupuncture has been widely used in the treatment of PSUI. Currently, many studies have shown that acupuncture is effective for PSUI. There are researches suggesting that acupuncture may improve symptoms of stress urinary incontinence by facilitating the reinnervation and strengthening of pelvic floor muscles.^[18–20]^ A previously published meta-analyses concluded that electroacupuncture (EA) was superior to sham EA or no intervention.^[21]^ Until now, no well-established systematic review has focused exclusively on acupuncture for PSUI. Accordingly, the first systematic review and meta-analysis will be conducted to investigate the efficacy and safety of acupuncture in the treatment of PSUI.

## 2. Methods

### 2.1. Study registration

This study protocol has been funded through a protocol registry. The registry number is INPLASY202220045, any revisions to the program will be documented on the INPLASY platform (https://inplasy.com/).

### 2.2. Criteria for considering studies

#### 2.2.1. Types of studies.

Only randomized controlled trials (RCTs) of acupuncture for the treatment of PSUI will be included, the language of articles included by our team will be limited to Chinese and English.

#### 2.2.2. Types of participants.

Participants will include patients diagnosed with PSUI based on The International Consultation on Urological Diseases without any age and race limit.^[22]^

#### 2.2.3. Types of interventions.

The interventions under consideration must involve needle insertion at acupuncture points, pain points, or trigger points, and have to be described as acupuncture. Studies evaluating the following treatments, including body acupuncture (manual acupuncture or EA), auricular acupuncture, scalp acupuncture, warm needle acupuncture, plum blossom needling, and fire needling, will be considered.

#### 2.2.4. Types of comparator(s)/control.

The inclusion of the comparator mainly included sham or placebo acupuncture intervention such as nonpenetrating, sham needle, or superficial needling at nonacupuncture points, moxibustion, massage, western medicine, pelvic floor muscle exercise, and bioelectrical stimulation therapy will also be taken into account.

### 2.3. Outcome measures

#### 2.3.1. Primary outcome.

The changes in pelvic floor muscle strength compared with baseline will be used as primary outcomes.

#### 2.3.2. Secondary outcomes.

The secondary outcomes will be the International Consultation on Incontinence Questionnaire-Urinary Incontinence Short Form score, the urodynamic indexes, the incontinence quality of life questionnaire, and acupuncture adverse events.

#### 2.3.3. Search strategies.

Firstly, RCTs of acupuncture treatment for PSUI were retrieved from PubMed, Web of Science , EMBASE, the Cochrane Central Register of Controlled Trials , Chinese National Knowledge Infrastructure , Chinese Biomedical Literature Database , Wanfang Database and Technology Periodical Database . We will consider articles published in English or Chinese between database initiation and December 2021. The studies will be independently retrieved by 2 researchers. According to different databases, we combine keywords and free words to conduct a comprehensive search. Medline's search strategy is shown in Table 1.

**Table 1:** The search strategy used in PubMed database.

NO.	Search items
#1	Randomized controlled trial [pt]
#2	Controlled clinical trial [pt]
#3	Randomized [tiab]
#4	Placebo [tiab]
#5	Clinical trials [MeSH]
#6	Randomly [tiab]
#7	Trial [ti]
#8	#1 OR #2 OR #3 OR #4 OR #5 OR #6 OR #7
#9	Humans [MeSH]
#10	#8 AND #9
#11	postpartum [MeSH]
#12	(Postnatal OR after delivery OR after childbirth) [ti, ab]
#13	#11 OR #12
#14	Stress urinary incontinence [MeSH]
#15	(Urinary Stress Incontinence OR Incontinence, Urinary Stress OR Stress Incontinence, Urinary) [ti, ab]
#16	#14 OR #15
#17	Acupuncture therapy [MeSH]
#18	Acupuncture OR Electroacupuncture OR (Electroacupuncture therapy) OR (body acupuncture) OR Electro-acupuncture OR (Manual acupuncture) OR (Auricular acupuncture) OR (Acupuncture and Moxibustion) OR (warm needling) [ti, ab]
#19	#17 OR #18
#20	#10 AND #13 AND #16 AND #19

Besides, the WHO International Clinical Trial Registry Platform, Chinese Clinical Trial Registry , ClinicalTrials.gov, Google scholar will be searched for any relevant ongoing or unpublished trials. The authors will also manually retrieve the relevant conference papers to obtain relevant information.

### 2.4. Data collection and analysis

#### 2.4.1. Selection of studies.

**Figure 1.**
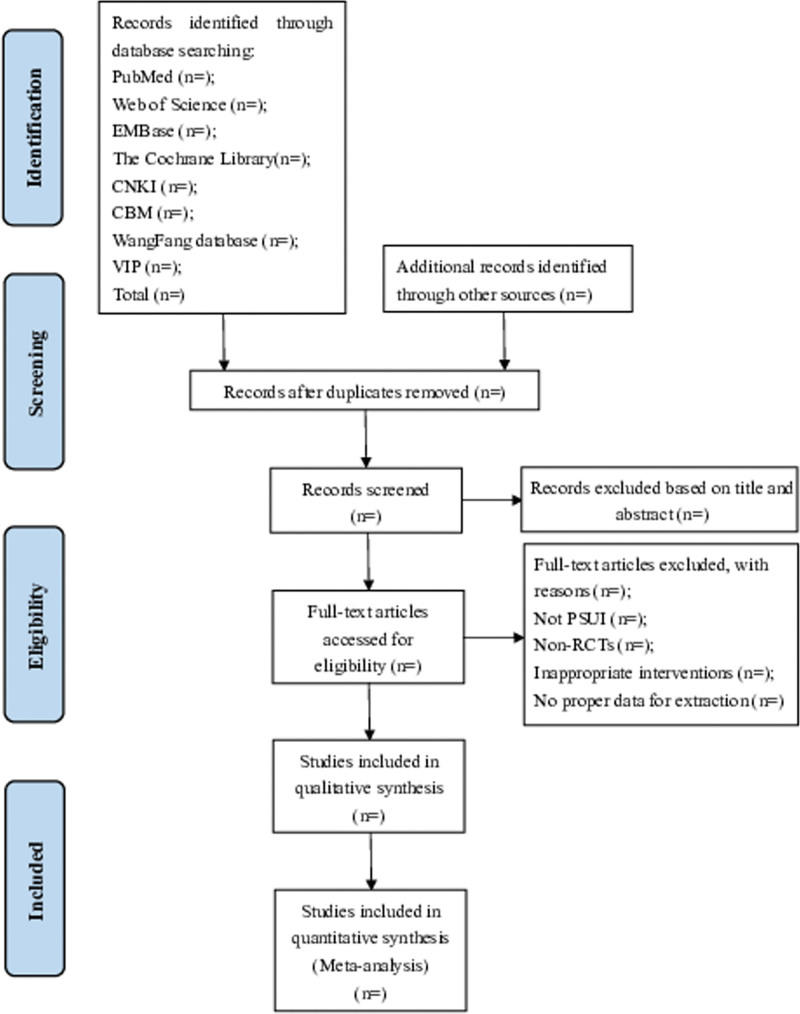
Flow diagram of the study selection process.

Two independent researchers will import the retrieved literature into NoteExpress 3.5.0 software literature management system according to the selected topic. First of all, we will delete the duplicate studies. Then, according to our established inclusion and exclusion, reliminary screening will be carried out by screening title, abstract and keywords to exclude irrelevant literature. The researchers will then read the full text and re-evaluate it. If there is any objections, it will be addressed by a third researchers. Besides, the exclusion of items and the reasons for exclusion will be recorded. The flow chart of literature screening is shown in Figure 1.

#### 2.4.2. Data extraction and management.

1. Two researchers will conduct data extraction and data management from the database. If there is data uncertainty, it will be solved through group discussion, and further differences will be solved by contacting authors and/or third-party arbitration. The basic information which will be extracted form the final included study will include:
2. First authors, years of publication, sources/journals, countries, and regions.
3. Subject characteristics such as sample size, age, diagnostic criteria, course of disease, severity of disease.
4. Inclusion and exclusion criteria, outcome indicators, interventions, research results, adverse reactions, and other detailed information.

#### 2.4.3. Risk of bias (quality) assessment.

The bias risk assessment included in the literature will be completed by 2 researchers through the Cochrane Collaboration's tool.^[23]^ The project covers: random sequence generation, allocation concealment, blinding methods for patients, researchers, and outcome evaluators, incomplete result data, selective reporting, and other prejudices. The evaluation results are divided into three levels: “low bias risk,” “high bias risk,” or “unclear bias risk.” Any differences will be resolved by discussion within the group or by a third researcher.

#### 2.4.4. Measures of treatment effect.

Review Manager (RevMan 5.3) software will be applied to perform statistical analysis. For continuous results, the mean difference or standardized mean difference will be used to evaluate the measurement data. For dichotomous outcomes, a risk ratio with 95% confidence intervals will be used for analysis.

#### 2.4.5. Dealing with missing data.

As for the missing or inadequate data, the researchers will attempt to get information by contacting the corresponding author. We will exclude these studies if the missing data cannot be supplied by the corresponding author.

#### 2.4.6. Assessment of heterogeneity.

We will use RevMan 5.3 software to evaluate statistical heterogeneity by *I*^2^ statistical test. The fixed-effect model will be used if *I*^2^ < 50%. *I*^2^ > 50%, there is significant heterogeneity between studies, sensitivity analysis, and subgroup analysis will be performed from clinical and methodological perspectives to find possible reasons.

#### 2.4.7. Assessment of reporting bias.

If more than 10 studies are included, the funnel plot will be used to construct the reporting bias. Otherwise, we will use the STATA 15.1 software to assess reporting bias by Egger test.

#### 2.4.8. Data synthesis.

The RevMan 5.3 will be carried out for data analysis and synthesis. We will use the fixed-effects model if there is little statistical heterogeneity between studies. Otherwise, the random-effects model will be used. We will carry out descriptive analysis if the results between studies are unsuitable to be combined.

#### 2.4.9. Subgroup analysis.

If necessary, subgroup analyses will be conducted based on the course of disease, severity of PSUI, different acupoints and therapy of the acupuncture, and course of treatment.

#### 2.4.10. Sensitivity analysis.

Sensitivity analyses will be carried out to test the robustness and reliability of the study conclusions. Decision nodes, including sample size, missing data, method quality will be considered. We will present the results of sensitivity analysis in summary tables.

#### 2.4.11. Quality of evidence evaluation.

The evidence quality of each outcome will be assessed by 2 researchers through the Grading of Recommendations Assessment, Development, and Evaluation system method independently.^[24]^ Then we will rate it into “very low,” mean difference low,” mean difference moderate,” or mean difference high” 4 levels to assess the quality of evidence.

## 3. Discussion

PSUI is a major public health problem that significantly affects postpartum women both in physical and mental health.^[8–12,25]^ Although surgery, medications and PFMT can reach a certain curative effect, there are shortcomings like difficult training techniques, adverse reaction of drugs and surgery.^[3,13–16]^ Acupuncture is a treatment method with fast curative effect and small side effect, which can improve PSUI symptoms by promoting nerve reinnervation, enhancing pelvic floor muscle strength and improving muscle coordination.^[18–20]^ Hitherto, there is still lack of valid evidence to support that acupuncture is effective for PSUI. Therefore, we need to perform a Systematic Review and Meta-analysis by including high-quality and up-to-date studies to assess the efficacy and safety of acupuncture for PSUI. We hope this study will provide robust evidence to inspire clinicians to conduct large-sample RCTs and promote the utilization of acupuncture for PSUI. Of course, there may be some potential limitations in this review. Specifically, due to the variance of acupuncture methods and the different severity of PSUI, the statistical heterogeneity may be higher. In addition, the quality of RCTs may be low, which will result in a risk of bias. The SR will be conducted and reported in strict accordance with the standards in the AMSTAR 2.0 and PRISMA. In the future, we will make further improvements and deepening of this study according to the deficiencies mentioned above and the actual changes.

## Acknowledgments

The authors would like to express their gratitude to all the advisors of this study.

## Author contributions

Conceptualization: Fengye Cao.

Data curation: Shanshan Zhang, Jingmei Huang, Fengye Cao.

Formal analysis: Fengye Cao, Shanshan Zhang.

Funding acquisition: Xianming Lin.

Methodology: Fengye Cao.

Resources: Fengye Cao.

Software: Fengye Cao, Lin Gan, Qinshuai Zhuansun.

Writing – original draft: Fengye Cao, Jingmei Huang.

Writing – review & editing: Fengye Cao, Shanshan Zhang, Jingmei Huang, Xianming Lin.
